# Italian translation of the Nine-Item ARFID Screen (NIAS-IT) for ARFID surveillance in a dietetic service

**DOI:** 10.1007/s40519-025-01807-3

**Published:** 2026-01-14

**Authors:** Lorenzo Casati, Tiziano Prodi, Anna Vedani, Camilla Gesi, Carmen Caruso, Anna Boggio, Bernardo Dell’Osso

**Affiliations:** 1 Department of Biomedical and Clinical Sciences “Luigi Sacco”, University of Milan, Milan, Italy; 2 Department of Psychiatry and Addiction ASST Fatebenefratelli-Sacco, Milan, Italy; 3 IUSS Cognitive Neuroscience (ICoN) Center, Scuola Universitaria Superiore IUSS, Pavia, Italy; 4 Clinical Nutrition and Dietetic, Luigi Sacco University Hospital, ASST Fatebenefratelli Sacco, Milan, Italy; 5 Department of Health Sciences, “Aldo Ravelli” Center for Neurotechnology and Brain Therapeutic, University of Milan, Milan, Italy; 6 “Centro per lo studio dei meccanismi molecolari alla base delle patologie neuro-psico- geriatriche”, University of Milan, Milan, Italy

**Keywords:** Avoidant/restrictive food intake disorder, ARFID, Dietary intervention, NIAS, Screening, Questionnaire, Eating disorder

## Abstract

**Background:**

Several versions of the Nine-Item ARFID Screen (NIAS) have been developed in recent years to adapt the questionnaire to different languages and clinical samples. An Italian version is still lacking. From the perspective of a highly specialized Eating Disorder Unit in Milano, ARFID’s phenomenology may be mimicked by other peculiar dietary habits or other eating disorders. Screening for this pathological conduct is necessary to assess a correct treatment frame for potentially serious disordered feeding behavior.

**Methods:**

Clinical experts in Psychiatry, Psychology, and Dietetics collaborated in the translation process. The whole methodology involved several steps: (I) Italian translation; (II) backward translation from Italian to English; (III) assessing the conformity between the original English and retranslated questionnaires; (IV) testing the translated version on patients; (V) evaluating the degree of comprehensibility of the translated scale.

**Key points:**

The Italian version of the NIAS (NIAS-IT) was administered online to 23 consecutive outpatients of a Dietetic department. For the most part, the sample of Italian native speakers found the translated version of the questionnaire to be comprehensible and easy to read.

**Conclusions:**

The Italian version of the NIAS is perfectly comprehensible and can be applied to the Italian population for both clinical and research purposes.

*Level of Evidence*: Level IV, evidence obtained from multiple time series with or without intervention.

## Introduction

Avoidant/restrictive food intake disorder (ARFID) is a multifaceted eating disorder whose phenomenology comprises three different drives for selective or restrictive feeding: reduced appetite, aversion to certain kinds of palatable food, or fear of negative consequences of eating [1]. The common outcome for this disorder is inadequate feeding, which can represent a cause of growth retardation in children, low body mass index (BMI), and anorexic-like appearance in adults, dependence on enteral feeding or oral supplementation, severe micronutrient deficiency, and/or impaired psychosocial functioning [2–4].

ARFID was for a long time considered a disorder mainly pertaining to the developmental age, despite the longitudinal outlook on eating behavior. Only recently, with the inclusion among Feeding and Eating Disorders (FED) in DSM-5 [5], ARFID effectively became a disorder that may span the entire life. Still, observations coming from the adult population are lacking [6]. While no studies on ARFID prevalence in Italy are available, data coming from a reference center in Rome showed a 65% increase in ARFID incidence from 2019 to 2024 [7].

Although the diagnosis is clinically based, screening instruments are being largely used to select subjects at high risk of FED within psychiatric and medical populations [8, 9]. ARFID has been especially found to be highly prevalent among subjects with neurodevelopmental disorders, with a notable comorbid pattern with autism spectrum disorders during childhood and adulthood [10, 11]. Moreover, several gastrointestinal conditions and diseases have been associated with ARFID, such as gastroesophageal reflux, eosinophilic esophagitis, and disorders of the gut–brain axis [12–14].

To set appropriate dietary regimens, subjects with such conditions are often referred to Clinical Nutrition and Dietetics Services, that also serve patients with immuno-dysmetabolic conditions that require specific exclusion diets, such as phenylketonuria, glycogenosis, migraine, irritating bowel syndrome (IBS), eosinophilic esophagitis, and allergic diathesis [15, 16]. It is important to note that exclusion diets may, in general, heighten the risk for eating disorders such as ARFID and that a pre-existing ARFID condition may suffer detrimental effects if the food avoidance is expanded or prolonged [13]. Therefore, patients referred to a Clinical Nutrition and Dietetics service might benefit from being screened for ARFID to avoid the severe consequences of malnutrition that can arise in the eventual dietetic prescription in already restricted or highly selective eating behaviors.

The Nine-Item ARFID Screen (NIAS) is a self-report questionnaire covering the three drives known to be responsible for restrictive/selective eating behaviors [17]. Originally developed as a self-report screening instrument for adult subjects [17] and subsequently as a parents-/caregivers-report on their children’s eating behaviors, the NIAS has been lately validated for children also [18, 19] and is widely used for ARFID screening. To the best of our knowledge, the NIAS has been already translated and culturally validated in several languages, including French, Turkish, Spanish, Arabic, and Chinese [20–24] and in Polish for the parent-reported version [25], while an Italian version of the scale is not currently available. All the cited studies showed that the translation of the NIAS in other languages and its cross-cultural adaptation did not affect its degree of comprehensibility and readability. Moreover, the reliability of the three-factor structure was conserved in the derived scales with respect to the original NIAS in terms of consistency: Cronbach’s α was 0.86 in the Chinese version [24], 0.88 in both the Spanish and Polish versions [22, 25], and 0.84 in the Arabic one [23], compared to the 0.90 in the original work [17]. Item-total correlations, distinguishing features between the items, and test–retest reliability were also proved to be maintained [21, 24].

The present study was thus aimed at reporting the structured translation of the NIAS from English to Italian and at providing clinicians and researchers with the Italian version (NIAS-IT) to be subsequently validated.

## Materials and methods

The original instrument adopted to begin the translational process is the English version [17]. The team of colleagues enrolled in the process comprised clinical experts in psychiatry and psychology (L.C., T.P., A.V., C.G.) and dietetics (A.B.) from the Eating Disorders Unit and Clinical Nutrition at the Fatebenefratelli-Sacco Hospital in Milano, Italy. The final version of the scale was lately administered via an online survey to 23 consecutive outpatients referred to the Clinical Nutrition service at the Fatebenefratelli-Sacco Hospital at the end of the routinary follow-up visit. The inclusion criterion was to be Italian mother-tongue speakers; the exclusion criterion was a diagnosis of any FED. The study was conducted in accordance with the declaration of Helsinki [26]. Patients provided their written informed consent to participate in the study.

### Structures and roles

The roles were assigned before the opening of the translational process [27]. C.G. supervised the whole process and communication between team members. Three translators (L.C., T.P., A.V.), Italian native speakers and fluent in English, separately transformed the nine questions into Italian. A.B. combined the three different Italian proposals into a single temporary version, which has been retranslated in English by an Italian native speaker fluent in English and academic writing (C.G.). Two independent reviewers (C.C., B.D.) compared the three initial proposals, the comparative draft, and the backtranslation to conclude the process.

### Translational procedure

Before we started with the translation, we made sure to contact the copyright owner so that we could obtain permission to proceed. Subsequently, we followed the guidelines described by Hernández and colleagues to ensure the best quality for the translational process [27]. The procedure started with three separate and individual translations of the English version of the NIAS into Italian. The three contributions were evaluated, compared, and combined into a single Italian draft: differences in the translations were compared, even if subtle, by clinicians expert in the field and fluent in English, as the plainness of the text was considered fundamental for a screening questionnaire. Table 1 shows an example of the differences between the three contributors. The initial Italian draft was then translated back to English. The result of the backtranslation was confronted with the original, with a good convergence and no need for further adjustments. Therefore, a pre-conclusive draft of the Italian NIAS questionnaire (NIAS-IT) was evaluated and confirmed as comprehensible by clinical experts in eating disorders and clinical research. The NIAS-IT had been administered via an online survey to patients referred to a Clinical Nutrition and Dietetics service in Milano. Patients completed a form requiring anthropometric measures, the completion of the NIAS-IT, and a separate questionnaire on the comprehensibility of the nine questions with a 5-point Likert scale (unintelligible, hard to understand, not too hard to understand, almost immediately understandable, and easily understandable) appositely developed. No identifiable information was gathered from participants. The degree of comprehensibility of the NIAS-IT was assessed by the result of the evaluation form.

**Table 1:** Shows the discrepancy between the three original and the final translation

“Non apprezzo la maggior parte dei cibi che mangiano gli altri”	“La maggior parte del cibo che mangiano le altre persone non mi piace”	“Non mi piacciono la maggior parte dei cibi che le altre persone mangiano”
Item 2: “Non mi piace la maggior parte dei cibi che mangiano le altre persone”		

## Results

The sample obtained by the answer to the online survey was made by 23 consecutive outpatients who had been visited by a clinician expert in dietetics in the service involved. The median age was 32 years [IQR: 28.50–48.50], and 16 (69.57%) were females; mean BMI was 22.01 (20.16 ± 2.10 standard deviation). The NIAS-IT mean total score was 12.56 (± 3.2) with a range of 9 (max 18, min 9); the single subscales scored under the cutoff, as expected in a general population sample (picky eating 5.30 ± 2.61, appetite 3.65 ± 1.69 and fear 3.60 ± 1.07, respectively). The totality of the sample (23/23, 100%) rated the questionnaire as “easy to understand”, with more than half (14/23) reporting an agreement ≥ 97.2%. Ten participants answered that the questionnaire was perfectly comprehensible with no degree of ambiguity; thirteen reported some degree of ambiguity, but not so intense as to hinder the correct comprehension of the questions. In detail, as shown by Table 2, all items were rated at least “almost immediately understandable” by all participants; just item 5 was rated by one participant as “hard to understand”, by another “not too hard to understand” and by a third one “almost immediately understandable”, with the rest of the sample (20/23, 86.9%) rating it as “easy to understand”.

**Table 2:** Shows the frequencies and percentages of the sample and their ratings over the nine items of the NIAS-IT scale. Possible rating options were “unintelligible”, “hard to understand”, “not too hard to understand”, “almost immediately understandable” and “easily understandable”

Item	Evaluation	*N* = 23 (%)
Item 1	Easily understandable	21 (91.3)
	Almost immediately understandable	2 (8.7)
Item 2	Easily understandable	18 (78.3)
	Almost immediately understandable	5 (21.7)
Item 3	Easily understandable	15 (65.2)
	Almost immediately understandable	8 (34.8)
Item 4	Easily understandable	21 (91.3)
	Almost immediately understandable	2 (8.7)
Item 5	Easily understandable	20 (87.0)
	Almost immediately understandable	1 (4.4)
	Not too hard to understand	1 (4.4)
	Hard to understand	1 (4.4)
Item 6	Easily understandable	18 (78.3)
	Almost immediately understandable	5 (21.8)
Item 7	Easily understandable	22 (95.7)
	Almost immediately understandable	1 (4.4)
Item 8	Easily understandable	22 (95.7)
	Almost immediately understandable	1 (4.4)
Item 9	Easily understandable	23 (100.0)

## Discussion

The administration of the NIAS-IT to a sample of outpatients referred to a Dietetics and Clinical Nutrition proved that the instrument is easy to understand and therefore could be used in the Italian population. The purpose of this work was to provide a manageable instrument for ARFID screening in Italian clinical settings pertaining to Clinical Nutrition and Dietetics or Eating Disorder Units. Unlike psychiatrists and psychologists, clinical experts in Dietetics may lack a specific formation in ARFID diagnosis, so providing an Italian version of the NIAS may increase the chance for a correct detection of this eating misconduct. Indeed, several organic disorders require elimination diets, which can lead to the development of a secondary ARFID or may worsen a pre-existing undiagnosed selective/restrictive eating behavior [13]. Elimination diets, usually prescribed for food allergies or intolerance, such as celiac disease (gluten-free diet), chronic kidney disease (low sodium and low proteins), or gastrointestinal pathologies such as IBS (low FODMAPS) or eosinophilic esophagitis (combined exclusion diet), are especially prone to this evolution in several cases [28–30], making ARFID surveillance important and useful. Thus, elimination diets should be carefully weighed and prescribed under strict medical supervision for the shortest period needed, especially in patients presenting selectivity traits, high among those suffering from gastrointestinal diseases such as IBS or gastroesophageal reflux disease [31]. The availability of an easily understandable screening tool is expected to widen the net of possible diagnoses for subthreshold disorders. It is worth noting that the NIAS is sufficient to orient the clinical suspicion if associated with another eating disorder scale such as the EDE-Q that scores negative, making it manageable even for non-clinical experts [9]. The use of this instrument will lead to more correct clinical classification of these patients showing or developing ARFID comorbidity, supporting the development of appropriate dietary regimens [32]. Moreover, the correct diagnosis of the comorbidity may help in delivering the dietary prescription with an adequate psychological frame and oriented communication. Nevertheless, literature on this topic is still in its initial stages, given that the formalization of the disease dates back just to 2013 [5]. This work’s secondary aim is to sensitize clinical professionals to the presence of a recently introduced diagnosis which can lead to severe consequences if undetected [4]. In conclusion, this work provided a useful instrument in Italian (NIAS-IT, Table 3) to screen for ARFID that may be relevant in Clinical Nutrition and Dietetics services. Similarly, it opens the path for the consideration of a novel Feeding and Eating Disorder in a country in which up to 51% of the general population, according to a recent national insurance investigation [33], is on a dietary regimen or pays particular attention to what and how much to eat.

**Table 3:** The definitive Italian version of the NIAS (NIAS-IT)

	Assolutamente in disaccordo (1)	In disaccordo (2)	Nè d’accordo nè in disaccordo (3)	D’accordo (4)	Assolutamente d’accordo (5)
Sono schizzinoso/a nell’alimentazione					
Non mi piace la maggior parte dei cibi che mangiano le altre persone					
La lista dei cibi che mi piacciono o che mangerei è più corta di quella dei cibi che non mangerei					
Non sono molto interessato/a a mangiare; sembra che io abbia meno appetito rispetto alle altre persone					
Devo sforzarmi di mangiare pasti regolari durante la giornata o di mangiare una quantità sufficiente di cibo ai pasti					
Anche quando mangio un cibo che mi piace molto, mi è difficile mangiarne una quantità adeguata durante i pasti					
Evito o rimando il momento di mangiare perché temo l’insorgere di fastidio gastrointestinale, soffocamento o vomito					
Mi limito a determinati alimenti perché temo che altri possano causarmi fastidio gastrointestinale, soffocamento o vomito					
Mangio piccole porzioni di cibo perché ho paura di provare un fastidio gastrointestinale, di soffocare o di vomitare					

The table shows the NIAS-IT as it appears in its original display. The three subtypes are listed from the top, with the appetite subscale first (item 1–3), the picky eating in second place (item 4–6) and the fear subscale in last position (item 7–9). Each item represents a Likert scale ranging from 1 to 5, for a maximum score of 15 for each subscale. We have adopted the cutoff already used by the developers to consider the screening positive: ≥ 9 for the appetite subscale, ≥ 10 for the picky eating subscale and ≥ 10 for the fear subscale

## Strength and limits

The main strength of this study is the rigorous and structured translation procedure, which followed international guidelines and was conducted by experts in psychiatry, psychology, and dietetics. This ensured both high linguistic accuracy and strong clinical appropriateness of the NIAS-IT. Moreover, the pilot testing on Italian-speaking outpatients confirmed excellent comprehensibility of all items. However, psychometric properties such as reliability, validity, sensitivity, and specificity were not evaluated and remain to be tested in future studies. The recruiting is still open for this objective. Future testing in different settings is an option, as ARFID cases may be detected even by general practitioners or within evaluations in sports centers. Moreover, the absence of participants with current or past FED limits the possibility of verifying how the NIAS-IT is actually interpreted by clinical individuals, for whom the tool is intended as a screening aid. Therefore, future research should include clinical samples to further test the discriminant validity and applicability of the NIAS-IT in diagnostic contexts. All this further evaluation of the NIAS-IT will provide a sounder instrument to screen ARFID in both clinical and non-clinical settings.

## What is already known on the subject?

Previous research has shown that the Nine-Item ARFID Screen (NIAS) is a widely used and reliable tool for ARFID screening across multiple languages such as French, Turkish, Spanish, Arabic, Chinese, and Polish, with a consistent factor structure and good internal reliability. However, an Italian version of the NIAS was still missing, leaving clinicians in Italy without a standardized, accessible tool for ARFID screening.

## What does this study add?

This study provides the first Italian translation of the NIAS (NIAS-IT) and proves its high comprehensibility for Italian-speaking adults. It offers clinicians and researchers an accessible tool to support ARFID screening in Italy and opens the path for future validation studies. The availability of the NIAS-IT may improve early identification of ARFID in medical and nutritional settings, supporting appropriate dietary and clinical management.

## Data Availability

Data will be made available upon request.

## References

[1] Calisan Kinter R, Ozbaran B, Inal Kaleli I, Kose S, Bildik T, Ghaziuddin M (2024) The sensory profiles, eating behaviors, and quality of life of children with autism spectrum disorder and avoidant/restrictive food intake disorder. Psychiatr Q 95(1):85–106. 10.1007/s11126-023-10063-6 38085408 10.1007/s11126-023-10063-6

[2] Sanchez-Cerezo J, Neale J, Julius N, Croudace T, Lynn RM, Hudson LD, Nicholls D (2024) Subtypes of avoidant/restrictive food intake disorder in children and adolescents: a latent class analysis. EClinicalMedicine 68:102440. 10.1016/j.eclinm.2024.102440 38333539 10.1016/j.eclinm.2024.102440 PMC10850399

[3] D’Adamo L, Smolar L, Balantekin KN, Taylor CB, Wilfley DE, Fitzsimmons-Craft EE (2023) Prevalence, characteristics, and correlates of probable avoidant/restrictive food intake disorder among adult respondents to the National Eating Disorders Association online screen: a cross-sectional study. J Eat Disord 11(1):1–15. 10.1186/s40337-023-00939-0 38049869 10.1186/s40337-023-00939-0 PMC10694964

[4] Schimansky S, Jasim H, Pope L, Hinds P, Fernandez D, Choleva P, Borman AD, Sharples PM, Smallbone T, Atan D (2023) Nutritional blindness from avoidant-restrictive food intake disorder—recommendations for the early diagnosis and multidisciplinary management of children at risk from restrictive eating. Arch Dis Child 109(3):181–187. 10.1136/archdischild-2022-325189 10.1136/archdischild-2022-325189 37414514

[5] American Psychiatric Association. Diagnostic and statistical manual of mental disorders (5th ed.). Arlington, VA: American Psychiatric Publishing, 2013

[6] MacDonald DE, Liebman R, Trottier K (2024) Clinical characteristics, treatment course and outcome of adults treated for avoidant/restrictive food intake disorder (ARFID) at a tertiary care eating disorders program. J Eat Disord 12(1):15. 10.1186/s40337-024-00973-6 38263130 10.1186/s40337-024-00973-6 PMC10807227

[7] Zanna Valeria, 2025; “Bambino Gesù” Hospital press release, 2025. https://img.ospedalebambinogesu.it/images/2025/03/10/113147825-1fe8d874-60e7-4ee3-99fd-201f598efa3e.pdf. Accessed 3 Sep 2025.

[8] Bryant-Waugh R, Stern CM, Dreier MJ, Micali N, Cooke LJ, Kuhnle MC, Burton Murray H, Wang SB, Breithaupt L, Becker KR, Misra M, Lawson EA, Eddy KT, Thomas JJ (2022) Preliminary validation of the pica, ARFID and rumination disorder interview ARFID questionnaire (PARDI-AR-Q). J Eat Disord 10(1):179. 10.1186/s40337-022-00706-7 36419081 10.1186/s40337-022-00706-7 PMC9682666

[9] Burton Murray H, Dreier MJ, Zickgraf HF, Becker KR, Breithaupt L, Eddy KT, Thomas JJ (2021) Validation of the nine item ARFID screen (NIAS) subscales for distinguishing ARFID presentations and screening for ARFID. Int J Eat Disord 54(10):1782–1792. 10.1002/eat.23520 33884646 10.1002/eat.23520 PMC8492485

[10] Menzel JE, Perry TR (2024) Avoidant/restrictive food intake disorder: review and recent advances. Focus (Am Psychiatr Publ) 22(3):288–300 38988468 10.1176/appi.focus.20240008 PMC11231462

[11] Casati L, Prodi T, Vedani A, Caruso C, Gesi C, Dell’Osso B (2024) The challenges of avoidant/restrictive food intake disorder and autism spectrum disorder comorbidity: a narrative update of course and outcomes. Curr Treat Options Psychiatry 11:358–365. 10.1007/s40501-024-00336-7

[12] Wronski M, Kuja-halkola R, Hedlund E, Martini MI, Lichtenstein P, Larsson H, Mark J, Micali N, Bulik CM, Dinkler L (2024) Co-existing mental and somatic conditions in Swedish children with the avoidant restrictive food intake disorder phenotype. medRxiv. 10.1101/2024.03.10.2430400338798326

[13] Weeks I, Abber SR, Thomas JJ, Calabrese S, Kuo B, Staller K, Murray HB (2023) The intersection of disorders of gut-brain interaction with avoidant/restrictive food intake disorder. J Clin Gastroenterol 57(7):651–662 37079861 10.1097/MCG.0000000000001853 PMC10623385

[14] Cifra N, Lomas JM (2022) Differentiating eosinophilic esophagitis and eating/feeding disorders. Pediatrics. 10.1542/peds.2021-052889 35243504 10.1542/peds.2021-052889

[15] Wei S, Li C, Wang Z, Chen Y (2023) Nutritional strategies for intervention of diabetes and improvement of β-cell function. Biosci Rep. 10.1042/BSR20222151 10.1042/BSR20222151 PMC9939408 36714968

[16] MacDonald A, van Wegberg AMJ, Ahring K, Beblo S, Bélanger-Quintana A, Burlina A, Campistol J, Coşkun T, Feillet F, Giżewska M, Huijbregts SC, Leuzzi V, Maillot F, Muntau AC, Rocha JC, Romani C, Trefz F, van Spronsen FJ (2020) PKU dietary handbook to accompany PKU guidelines. Orphanet J Rare Dis 15(1):171 32605583 10.1186/s13023-020-01391-y PMC7329487

[17] Zickgraf HF, Ellis JM (2018) Initial validation of the nine item avoidant/restrictive food intake disorder screen (NIAS): a measure of three restrictive eating patterns. Appetite 123:32–42. 10.1016/j.appet.2017.11.111 29208483 10.1016/j.appet.2017.11.111

[18] Carmody J, Milliren C, Richmond T, Crowley M, Eldredge O, Jhe G, Freizinger M, Bern E (2025) Caregiver-youth agreement on the nine-item avoidant/restrictive food intake disorder survey. Int J Eat Disord 58:770–777. 10.1002/eat.24384 39853817 10.1002/eat.24384

[19] Billman Miller MG, Zickgraf HF, Murray HB, Essayli JH, Lane-Loney SE (2023) Validation of the youth-nine item avoidant/restrictive food intake disorder screen. Europ Eat Disord Rev. 10.1002/erv.3017 10.1002/erv.3017 PMC10840920 37545024

[20] Van Ouytsel P, Mikhael-Moussa H, Mion F, Roman S, Gourcerol G, Marion-Letellier R, Piessevaux H, Louis H, Melchior C (2024) Translation of the nine item avoidant/restrictive food intake disorder screen (NIAS) questionnaire in French (NIAS-Fr). Neurogastroenterol Motil 36(4):e14757 38308088 10.1111/nmo.14757

[21] Öğütlü H, Kaşak M, Doğan U, Zickgraf HF, Türkçapar MH (2024) Psychometric properties of the nine-item avoidant/restrictive food intake disorder screen (NIAS) in Turkish children. J Eat Disord 12(1):30. 10.1186/s40337-024-00987-0 38374128 10.1186/s40337-024-00987-0 PMC10875749

[22] Medina-Tepal KA, Vazquez-Arevalo R, Trujillo-ChiVacuán EM, Zickgraf HF, Mancilla-Díaz JM (2023) Cross-cultural adaptation and validation of the nine item ARFID screen (NIAS) in Mexican youths. Int J Eat Disord 56(4):721–726. 10.1002/eat.23832 36268632 10.1002/eat.23832

[23] Fekih-Romdhane F, Hallit R, Malaeb D et al (2023) Psychometric properties of an Arabic translation of the Nine Item Avoidant/Restrictive Food Intake Disorder Screen (NIAS) in a community sample of adults. J Eat Disord 11:143. 10.1186/s40337-023-00874-0 37612764 10.1186/s40337-023-00874-0 PMC10463449

[24] He J, Zickgraf HF, Ellis JM, Lin Z, Fan X (2021) Chinese version of the Nine Item ARFID Screen: psychometric properties and cross-cultural measurement invariance. Assessment 28(2):537–550. 10.1177/1073191120936359 32608255 10.1177/1073191120936359

[25] Ziółkowska B, Ocalewski J, Zickgraf H, Brytek-Matera A (2022) The Polish version of the Avoidant/Restrictive Food Intake Disorder Questionnaire—Parents Report (ARFID-Q-PR) and the Nine Items Avoidant/Restrictive Food Intake Disorder Screen—Parents Report (NIAS-PR): maternal perspective. Nutrients 14(15):3175. 10.3390/nu14153175 35956354 10.3390/nu14153175 PMC9370130

[26] World Medical Association (2001) World Medical Association Declaration of Helsinki Ethical Principles for medical research involving human subjects. Bull World Health Organ 79(4):373–374 11357217 PMC2566407

[27] Hernández A, Hidalgo MD, Hambleton RK, Gómez-Benito J (2020) International Test Commission guidelines for test adaptation: a criterion checklist. Psicothema 32(3):390–398. 10.7334/psicothema2019.306 32711675 10.7334/psicothema2019.306

[28] Chey WD (2019) Elimination diets for irritable bowel syndrome: approaching the end of the beginning. Am J Gastroenterol 114(2):201–203 30676371 10.14309/ajg.0000000000000099

[29] Robson J, Laborda T, Fitzgerald S, Andersen J, Peterson K, O’Gorman M, Guthery S, Bennett-Murphy L (2019) Avoidant/restrictive food intake disorder in diet-treated children with eosinophilic esophagitis. J Pediatr Gastroenterol Nutr 69(1):57–60 30889128 10.1097/MPG.0000000000002323

[30] Fitzgerald M, Frankum B (2017) Food avoidance and restriction in adults: a cross-sectional pilot study comparing patients from an immunology clinic to a general practice. J Eat Disord 5(1):30 28936357 10.1186/s40337-017-0160-4 PMC5603184

[31] Malone JC, Daley SF (2024) Elimination Diets. StatPearls Publishing, Treasure Island 38261686

[32] Archibald T, Bryant-Waugh R (2023) Current evidence for avoidant restrictive food intake disorder: implications for clinical practice and future directions. JCPP Adv 3(2):e12160 37753149 10.1002/jcv2.12160 PMC10519741

[33] UniSalute. (n.d.). La metà degli italiani segue una dieta, ma pochi si affidano a uno specialista. www.unisalute.it. Accessed 4 Jan 2025.

